# Nonalcoholic fatty liver disease is an early predictor of metabolic diseases in a metabolically healthy population

**DOI:** 10.1371/journal.pone.0224626

**Published:** 2019-11-04

**Authors:** Seokhun Yang, Soongu Kwak, Jeong-Hoon Lee, Shinae Kang, Seung-Pyo Lee

**Affiliations:** 1 Division of Cardiology and Cardiovascular Institute, Department of Internal Medicine, Seoul National University Hospital, Seoul, South Korea; 2 Division of Gastroenterology and Liver Research Institute, Department of Internal Medicine, Seoul National University Hospital, Seoul, South Korea; 3 Division of Endocrinology, Department of Internal Medicine, Yonsei University, Seoul, South Korea; Policlinico Universitario Campus Bio-Medico, ITALY

## Abstract

**Aims:**

The relationship between nonalcoholic fatty liver disease and incident metabolic syndrome in metabolically healthy subjects is unknown. We aimed to investigate whether nonalcoholic fatty liver disease is a predictor of future metabolic syndrome in metabolically healthy subjects.

**Materials and methods:**

Subjects who underwent health evaluation at least twice between 2009 and 2015 from the National Health Insurance Service-National Sample Cohort in South Korea were included. Patients without obesity who had no metabolic syndrome components were finally analyzed (n = 28,880). The definition of nonalcoholic fatty liver disease was based on both the hepatic steatosis and fatty liver indices. The incidence of metabolic syndrome, prediabetes/type 2 diabetes, hypertension, and dyslipidemia was compared between the subjects with and without nonalcoholic fatty liver disease.

**Results:**

The presence of nonalcoholic fatty liver disease was associated with a higher risk of incident metabolic syndrome, prediabetes/type 2 diabetes, hypertension, and dyslipidemia in the entire cohort (metabolic syndrome: adjusted hazard ratio, 2.10; 95% confidence interval, 1.18–3.71; prediabetes/type 2 diabetes: adjusted hazard ratio, 1.42; 95% confidence interval, 1.06–1.90; hypertension: adjusted hazard ratio, 2.36; 95% confidence interval, 1.35–4.12; dyslipidemia: adjusted hazard ratio, 1.49; 95% confidence interval, 1.07–2.06). A similar finding was observed in the age-, sex-, smoking status-, and body mass index-based 1:5 propensity score-matched cohort of 1,092 subjects (metabolic syndrome: adjusted hazard ratio, 3.56; 95% confidence interval, 1.79–7.07; prediabetes/type 2 diabetes: adjusted hazard ratio, 1.97; 95% confidence interval, 1.04–3.73; hypertension: adjusted hazard ratio, 2.57; 95% confidence interval, 1.35–4.88; dyslipidemia: adjusted hazard ratio, 1.61; 95% confidence interval, 1.12–2.32).

**Conclusions:**

Nonalcoholic fatty liver disease is an early predictor of metabolic dysfunction even in metabolically healthy populations.

## Introduction

A bidirectional relationship between nonalcoholic fatty liver disease (NAFLD) and metabolic disease has recently been highlighted [1–5]. NAFLD may induce metabolic dysfunction through hepatic insulin resistance, chronic metabolic stressor production, and inflammatory response caused by hepatokines and lipid accumulation [6,7]. Several observational studies demonstrated that NAFLD precedes type 2 diabetes, hypertension, dyslipidemia, and metabolic syndrome [8–10]. However, previous studies are limited in providing evidence as to which condition comes first because NAFLD is usually accompanied with metabolic syndrome components.

Recently, the presence of NAFLD has been reported to be associated with an increased risk of metabolic dysfunction in patients with one or more metabolic syndrome components [11], which raises the issue of whether NAFLD is indeed a surrogate of metabolic disease in a population without metabolic syndrome. This issue is clinically relevant because NAFLD is common in the general population [12], and its association with cardiovascular risks not attributed to metabolic dysfunction has been reported [13,14]. If NAFLD is a surrogate of metabolic disease even in those without any metabolic syndrome components, it will provide a basis for starting an earlier intervention, such as stricter lifestyle modification.

In this regard, we hypothesized that the presence of NAFLD is an early phenotypic predictor of future metabolic dysfunction in a metabolically healthy population. By applying simple biochemical indices developed to predict the presence of NAFLD [15,16], we investigated the impact of NAFLD on the development of metabolic disease in a truly metabolically healthy population.

## Materials and methods

### Study design and participants

This study was based on the national health claims database provided by the National Health Insurance Service (NHIS) of South Korea. The NHIS is a mandatory national insurance service that covers more than 97% of all Koreans and provides biennial health examination to all participants. For the current analysis, we included study participants from the NHIS-National Sample Cohort (NHIS-NSC) version 2.0, which was updated from the NHIS-NSC in 2017 and a representative cohort including 1,108,369 participants randomly extracted from the total South Korean population. The detailed design and profile of the NHIS-NSC have been described previously [17]. Each participant’s demographic characteristics, diagnoses, and prescription records in both the inpatient and outpatient services are available in the NHIS-NSC. The claim diagnoses are based on the International Classification of Disease-10^th^ Revision-Clinical Modification (ICD-10-CM) codes. The overall study was approved by the Seoul National University Hospital institutional review board (E-1807-001-953). The need for informed consent was waived by the institutional review board as the information of the participants used for the analysis was anonymized and unidentified. The study protocol was in accordance with the ethical guidelines of the 1975 Declaration of Helsinki.

### Study population

**Fig 1:**
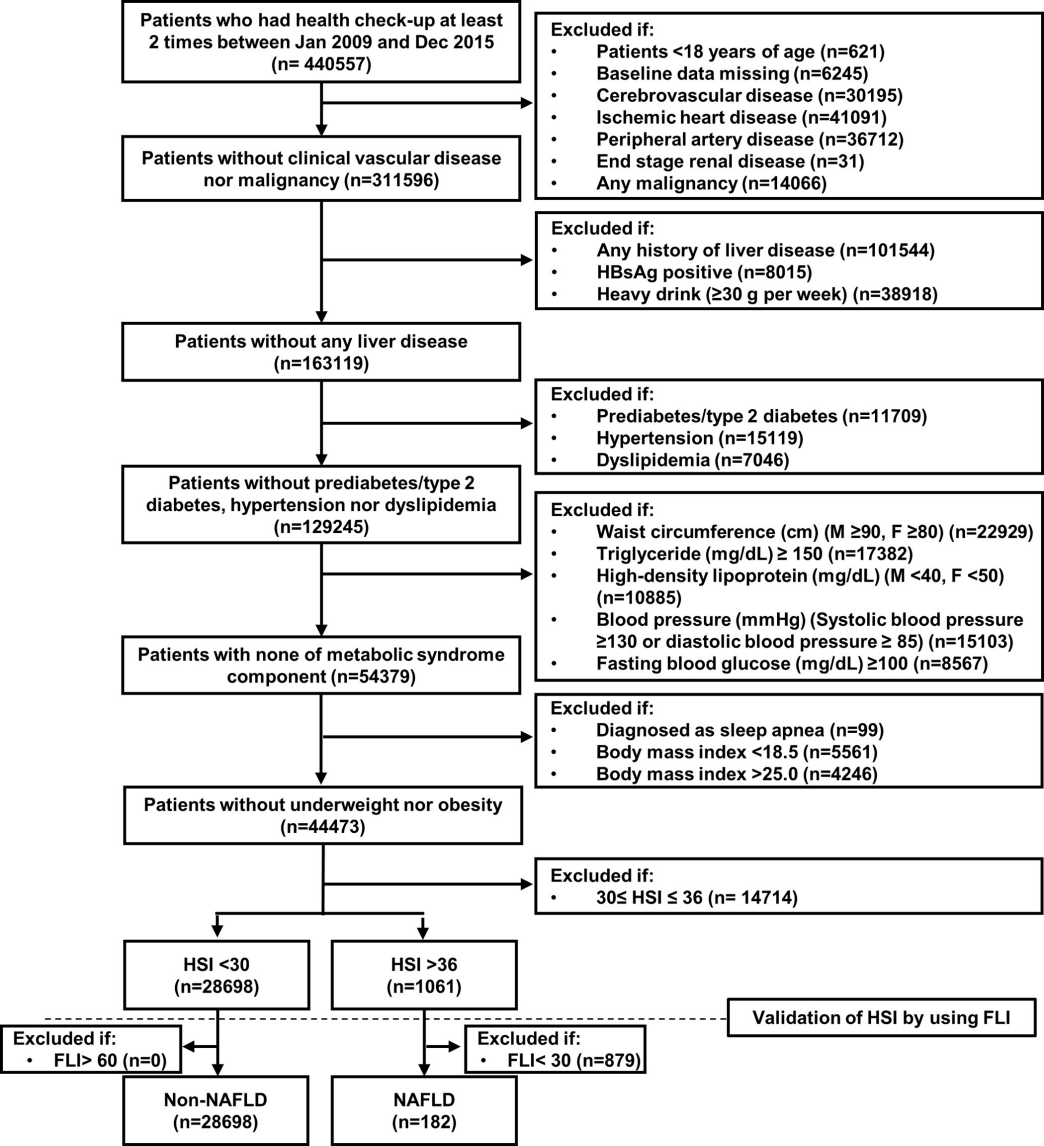
Flow chart and the selection of the study participants. FLI, fatty liver index; HSI, hepatic steatosis index; NAFLD, nonalcoholic fatty liver disease.

Participants aged ≥18 years who underwent health evaluation at least two times between January 2009 and December 2015 were included. The basis of including these participants was that at least two health evaluations were needed after a baseline evaluation to make a diagnosis of incident metabolic syndrome. To prevent potential bias by preceding cardiovascular events, malignancy, and other liver diseases, we excluded those with a history of cerebrovascular disease (ICD-10-CM codes I60–I64), ischemic heart disease (ICD-10-CM codes I22–I23), peripheral artery disease (ICD-10-CM codes I70 and I73), end-stage renal disease (ICD-10-CM codes N185 and Z49), any malignancy (ICD-10-CM codes C00–C97), and any liver disease (ICD-10-CM codes K70–K77), alcoholics (≥30 g per week), and those with positive hepatitis B antigen findings in the baseline health evaluation. We finally included metabolically healthy participants with non-obese body mass index (BMI, 18.5–24.9 kg/m^2^) after further excluding those who had a history of type 2 diabetes (ICD-10-CM codes E11–E14) or anti-diabetic medication use, hypertension (ICD-10-CM codes I10–I15) or anti-hypertensive medication use, dyslipidemia (ICD-10-CM codes E78 and its all sub-classifications) or lipid-lowering medication use, and sleep apnea (ICD-10-CM code G473) and no metabolic syndrome components as defined using the National Cholesterol Education Program Adult Treatment Panel III criteria [18,19] at baseline health evaluation (**Fig 1**).

### Definition of NAFLD

The hepatic steatosis index (HSI) is a validated index used to predict the presence of NAFLD; it has been found to have a high specificity and sensitivity in a large cohort of Koreans [15]. We divided the participants into two groups according to the HSI cutoff value of specificity and sensitivity: NAFLD group: HSI of >36, indicating high probability of NAFLD and non-NAFLD group: HSI of <30, indicating low probability of NAFLD. As the metabolically healthy study participants are less likely to have NAFLD than the entire general population, we cross-validated the presence of NAFLD using the fatty liver index (FLI), which is an index of proven predictability for the presence of NAFLD in the European population [16]. We applied a sensitivity cutoff FLI value of 30 to the group with an HSI of >36 to exclude false-positive HSI and a specificity cutoff FLI value of 60 to the group with an HSI of <30 to test the rate of false-negative HSI. Among the total 1,061 participants with an HSI of >36, 182 participants were considered to have NAFLD after further excluding 879 participants who had a low FLI (defined as false-positive HSI). As none of the participants with an HSI of <30 had a high FLI, a total of 28,698 participants were considered to have no NAFLD.

### Primary and secondary endpoints

The enrollment date was defined as the date of each participant’s first health evaluation between January 2009 and December 2014. The data cutoff date was December 31, 2015. The primary endpoint was incident metabolic syndrome during follow-up. Metabolic syndrome was defined using the National Cholesterol Education Program Adult Treatment Panel III criteria [18,19]: three or more of the following five criteria: waist circumference of >90 cm in men or >80 cm in women, blood pressure of >130/85 mmHg, fasting triglyceride (TG) level of >150 mg/dL, fasting high-density lipoprotein (HDL) cholesterol level of <40 mg/dL in men or <50 mg/dL in women, and fasting blood glucose level of >100 mg/dL.

The secondary endpoints were incident prediabetes/type 2 diabetes, hypertension, and dyslipidemia. Incident prediabetes was defined as a serum fasting glucose level of >100 mg/dL at follow-up health evaluation or a clinical diagnosis of prediabetes (ICD-10-CM code R73 or its all sub-classifications). Incident type 2 diabetes was defined as a clinical diagnosis of type 2 diabetes (ICD-10-CM codes E11–E14) or use of anti-diabetic medications. Incident hypertension was defined as a clinical diagnosis of hypertension (ICD-10-CM codes I10–I15) or use of anti-hypertensive medications and incident dyslipidemia as a clinical diagnosis of disorders of lipoprotein metabolism and other lipidemias (ICD-10-CM code E78 and its all sub-classifications) or use of lipid-lowering medications.

### Statistical analysis

Categorical variables were shown as numbers (percentages) and continuous variables as means ± standard deviations. The difference in the categorical variables between the groups was assessed using the chi-square test and that in the continuous variables using one-way analysis of variance or the Kruskal-Wallis test as appropriate. *P*-values of <0.05 were considered statistically significant. All analysis was performed using the R language version 3.4.3 (R Foundation for Statistical Computing, Vienna, Austria).

We performed a propensity score matching analysis to focus on the effect of NAFLD on the incidence of metabolic syndrome and to reduce the impact of differences in the demographics between the NAFLD and non-NAFLD groups. The propensity of being in the NAFLD group was calculated using a logistic regression model including age, sex, smoking status, and category of BMI (normal or overweight). The NAFLD group and the non-NAFLD group were matched at a ratio of 1:5 using the nearest-neighbor method without replacement, with a caliper of 0.01 of the propensity score. We applied univariate and multivariate standard Cox regression models to test whether the presence of NAFLD is an early predictor of the primary and secondary endpoints in the entire cohort and the propensity score-matched cohorts. In the multivariate analysis, age, sex, smoking status, and the category of BMI (normal or overweight) were included in the Cox regression model. We also performed a subgroup analysis based on age, sex, smoking status, and category of BMI (normal or overweight). A mediation analysis was performed using a logistic regression model to verify the direct effect of NAFLD on the primary and secondary endpoints.

## Results

### Baseline characteristics of the study participants

The baseline characteristics of the study participants before and after propensity score matching are shown in **Table 1**. After application of the inclusion and exclusion criteria, there were a total of 28,880 participants without obesity who had no metabolic syndrome components. At baseline evaluation, the NAFLD group subjects were older and more likely to be men, current smokers, and overweight. After 1:5 propensity score matching, a total of 1,092 patients were included in the final analysis (NAFLD group: n = 182; non-NAFLD group: n = 910). Among the propensity score-matched cohort, the mean age was 37.2 years and 37.4 years in the NAFLD and non-NAFLD groups, respectively (*P* = 0.68). The proportion of male subjects, current smokers, and overweight subjects was the same between the two groups. The total cholesterol, TG, HDL cholesterol, and low-density lipoprotein cholesterol levels and blood pressure were all in the normal range in both the NAFLD and non-NAFLD groups. Among the laboratory findings, only the alanine transaminase level was higher than the normal range in the NAFLD group.

**Table 1:** Baseline clinical characteristics of study population before and after propensity score matching, according to presence of NAFLD.

	Entire cohort (n = 28,880)			Matched cohort (n = 1,092)		
	Non-NAFLD (n = 28,698)	NAFLD (n = 182)	p-value	Non-NAFLD (n = 910)	NAFLD (n = 182)	p-value
Age (yrs)	36.0±10.9	37.4±8.9	<0.01	37.2±9.0	37.4±8.9	0.68
Male (n, %)	10237 (35.7)	167 (91.8)	<0.01	835 (91.8)	167 (91.8)	1.00
BMI						
Normal (n, %)	26782 (93.3)	8 (4.4)	<0.01	40 (4.4)	8 (4.4)	1.00
Overweight (n, %)	1916 (6.7)	174 (95.6)	<0.01	870 (95.6)	174 (95.6)	1.00
Current smoking (n, %)	5106 (17.8)	84 (46.2)	<0.01	420 (46.2)	84 (46.2)	1.00
Waist circumference (cm)						
Male	75.7±4.9	83.7±3.5	<0.01	79.8±4.2	83.7±3.5	<0.01
Female	68.4±4.5	77.1±1.6	<0.01	73.0±3.4	77.1±1.6	<0.01
Laboratory findings						
ALT (IU/L)	14.0±7.4	58.0±39.8	<0.01	14.9±6.8	58.0±39.8	<0.01
AST (IU/L)	20.4±12.6	31.2±17.1	<0.01	23.4±13.7	31.2±17.1	<0.01
ALT/AST ratio	0.7±0.2	1.8±0.4	<0.01	0.7±0.1	1.8±0.4	<0.01
GGT (IU/L)	16.8±9.6	72.7±47.6	<0.01	20.3±9.3	72.7±47.6	<0.01
Total cholesterol (mg/dL)	180.3±29.6	204.8±33.8	<0.01	182.4±29.7	204.8±33.8	<0.01
Triglyceride (mg/dL)	72.7±27.2	116.8±21.2	<0.01	83.5±28.5	116.8±21.2	<0.01
LDL-cholesterol (mg/dL)	110.5±24.3	127.8±32.8	<0.01	109.7±44.9	127.8±32.8	<0.01
HDL-cholesterol (mg/dL)	63.7±19.7	53.9±9.3	<0.01	57.4±18.5	53.9±9.3	<0.01
Fasting blood glucose (mg/dL)	86.1±7.6	88.2±7.2	<0.01	86.4±7.6	88.2±7.2	<0.01
Systolic blood pressure (mmHg)	110.6±9.3	115.5±7.5	<0.01	113.8±8.3	115.5±7.5	0.01
Diastolic blood pressure (mmHg)	69.3±7.3	72.8±6.4	<0.01	71.6±6.9	72.8±6.4	0.06
Creatinine (mg/dL)	1.0±1.2	1.1±1.2	<0.01	1.2±1.3	1.1±1.2	0.24
Interval between baseline and follow-up evaluation (years)	1.8±1.0	1.7±1.1	0.09	1.7±1.0	1.7±1.1	0.53
Clinical event						
Metabolic syndrome (n, %)	294 (1.0)	15 (8.2)	<0.01	18 (2.0)	15 (8.2)	<0.01
Prediabetes/Type 2 diabetes mellitus (n, %)	4581 (16.0)	49 (26.9)	<0.01	158 (17.4)	49 (26.9)	<0.01
Hypertension (n, %)	768 (2.7)	14 (7.7)	<0.01	28 (3.1)	14 (7.7)	<0.01
Dyslipidemia (n, %)	4362 (15.2)	38 (20.9)	0.04	122 (13.4)	38 (20.9)	0.01
Hepatic steatosis index	27.6±1.6	38.8±3.0	-	28.9±1.0	38.8±3.0	-
Fatty liver index	5.4±4.6	38.1±7.0	-	13.1±6.7	38.1±7.0	-

The data are presented as mean±SD for continuous variables and number (percentage) for categorical variables. ALT, alanine aminotransferase; AST, aspartate aminotransferase; BMI, body mass index; GGT, gamma-glutamyl transferase; HDL, high-density lipoprotein; LDL, low-density lipoprotein; NAFLD, nonalcoholic fatty liver disease.

### Higher incidence of metabolic syndrome in the NAFLD group

**Fig 2:**
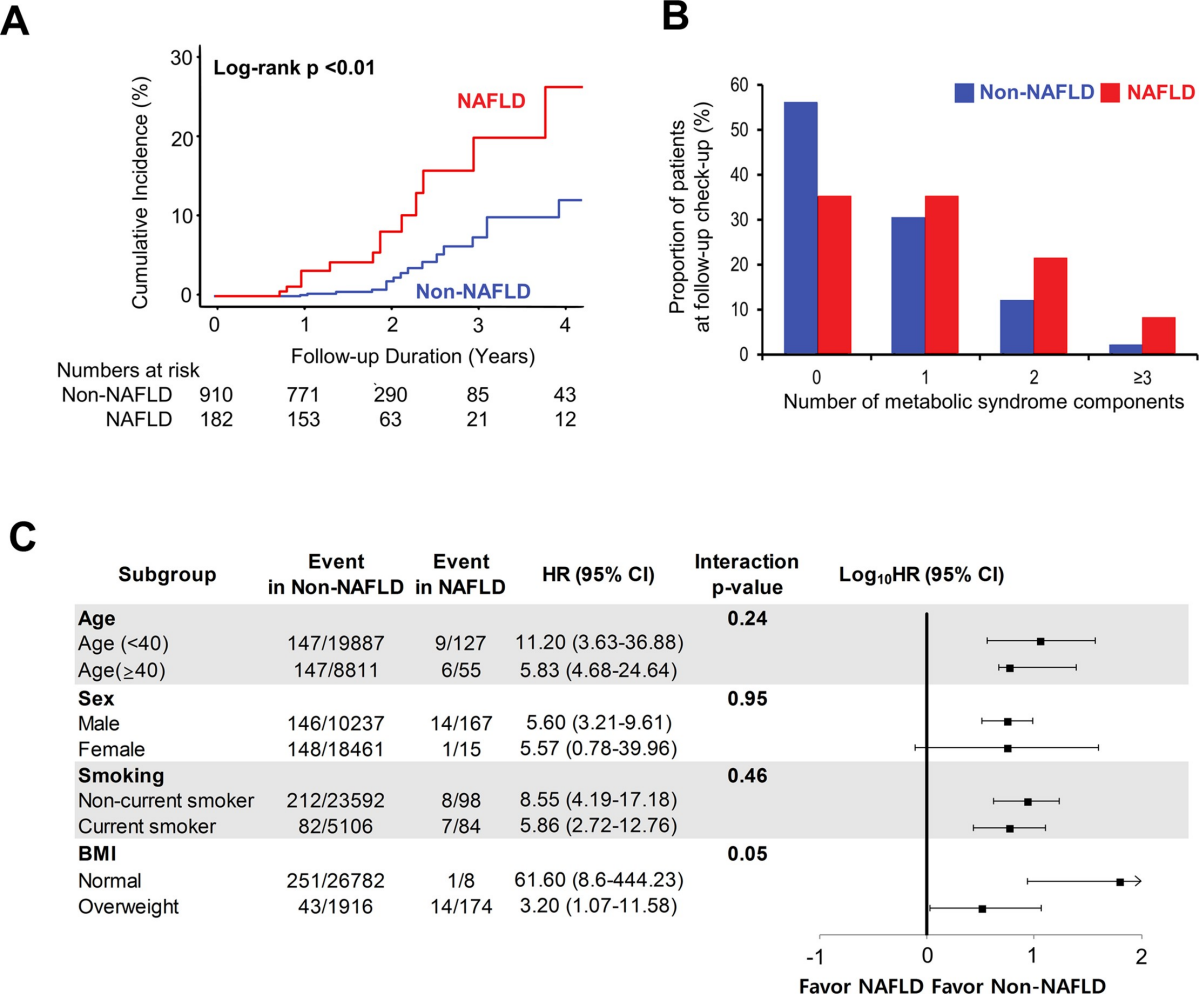
NAFLD as an independent early predictor of incident metabolic syndrome. **(A)** Cumulative incidence of metabolic syndrome in the propensity score matched cohort, according to the presence of NAFLD. **(B)** Proportion of patients with each number of metabolic syndrome components at follow-up health evaluation, according to the presence of NAFLD. **(C)** Subgroup analysis of incident metabolic syndrome. BMI, body mass index; CI, confidence interval; HR, hazard ratio; NAFLD, nonalcoholic fatty liver disease.

Among the entire metabolically healthy subjects (n = 28,880), the risk of incident metabolic syndrome at the follow-up health evaluation was significantly higher in the NAFLD group than in the non-NAFLD group (unadjusted hazard ratio (uHR), 8.60; 95% confidence interval (CI), 5.12–14.46, *P*<0.01; adjusted hazard ratio (aHR), 2.10; 95% CI, 1.18–3.71, *P* = 0.01). This result was similar in the propensity score-matched cohort (uHR 2.76, 95% CI, 1.59–4.81, *P*<0.01; aHR, 3.56; 95% CI, 1.79–7.07, *P<*0.01) (**Table 2**). The cumulative incidence of metabolic syndrome was significantly higher in the NAFLD group both before and after propensity matching (log-rank *P*<0.01 for the unmatched cohort, **S1 Fig**; log-rank *P*<0.01 for the propensity score-matched cohort, **Fig 2A**). The number and proportion of patients with each of the components of metabolic syndrome in both the NAFLD and non-NAFLD groups at follow-up evaluation demonstrated that the difference in the incidence of newly diagnosed metabolic syndrome between them was mainly because of the development of abdominal obesity, hypertriglyceridemia, and prediabetes (**S1 Table**). Although statistically insignificant, the proportion of subjects with incident high blood pressure and low HDL cholesterol level at follow-up was also higher in the NAFLD group. After propensity score matching, the proportion of participants without any metabolic syndrome component at follow-up was still significantly lower in the NAFLD group than in the non-NAFLD group (35.2% vs 55.9%, *P*<0.01); conversely, the proportion of participants with two or more metabolic syndrome components was higher in the NAFLD group (**S1 Table** and **Fig 2B**). The subgroup analysis also demonstrated that the effect of NAFLD on future metabolic syndrome was irrespective of age, sex, smoking status, and BMI (**Fig 2C**).

**Table 2:** Univariate and multivariate Cox proportional-hazards regression analysis for incident metabolic syndrome in the entire cohort and the propensity score-matched cohort.

	Total cohort (n = 28,880)				Matched cohort (n = 1,092)	
	Unadjusted		Covariate-adjusted^†^		Unadjusted	
	HR (95% CI)	p-value	HR (95% CI)	p-value	HR (95% CI)	p-value
NAFLD	8.60 (5.12–14.46)	<0.01	2.10 (1.18–3.71)	0.01	3.56 (1.79–7.07)	<0.01
Age	1.04 (1.03–1.04)	<0.01	1.03 (1.02–1.04)	<0.01	1.01 (0.97–1.05)	0.60
Male	2.50 (2.00–3.13)	<0.01	1.39 (1.06–1.84)	0.02	0.81 (0.24–2.66)	0.73
Body mass index	1.56 (1.45–1.69)	<0.01	1.40 (1.29–1.53)	<0.01	1.04 (0.14–7.62)	0.97
Current smoking	2.20 (1.74–2.84)	<0.01	1.60 (1.20–2.14)	<0.01	1.29 (0.65–2.55)	0.47

Cox proportional-hazards regression analysis to identify predictors of incident metabolic syndrome at follow-up health evaluation.

^†^, adjusted for NAFLD, age, male, body mass index, current smoking.

CI, confidence interval; HR, hazard ratio; NAFLD, non-alcoholic fatty liver disease.

### Relationship between NAFLD and incident prediabetes/type 2 diabetes, hypertension, and dyslipidemia

In the secondary endpoint analysis, the risk of incident prediabetes/type 2 diabetes, hypertension, and dyslipidemia was higher in the NAFLD group even after adjusting for age, sex, smoking status, and BMI (aHR for prediabetes/type 2 diabetes, 1.42; 95% CI, 1.06–1.90, *P* = 0.02; aHR for hypertension, 2.36; 95% CI, 1.35–4.12, *P*<0.01; aHR for dyslipidemia, 1.49; 95% CI, 1.07–2.06, *P* = 0.02). This result was similar in the propensity score-matched cohort (aHR for prediabetes/type 2 diabetes, 1.97; 95% CI, 1.04–3.73, *P =* 0.04; aHR for hypertension, 2.57; 95% CI, 1.35–4.88, *P*<0.01; aHR for dyslipidemia, 1.61; 95% CI, 1.12–2.32, *P = 0*.*01*) (**S2 Table**).

**Fig 3:**
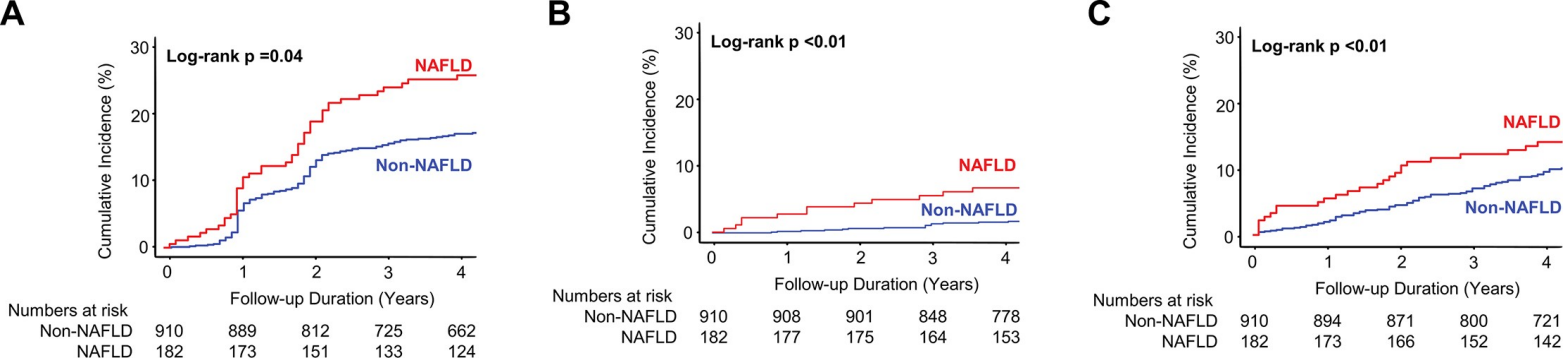
NAFLD as a possible surrogate of metabolic dysfunction. Cumulative incidence of **(A)** prediabetes/type 2 diabetes, **(B)** hypertension, and **(C)** dyslipidemia in the propensity score matched cohort, according to the presence of NAFLD. NAFLD, nonalcoholic fatty liver disease.

Among the entire cohort (n = 28,880), the cumulative incidence of prediabetes/type 2 diabetes, hypertension, and dyslipidemia was higher in the NAFLD group (**S2 Fig**). This result was consistent in the propensity score-matched cohort of 1,092 subjects (**Fig 3**).

A sensitivity analysis was performed in all subgroups according to age, sex, smoking status, and BMI. The trends for incident prediabetes/type 2 diabetes, hypertension, and dyslipidemia were consistent in all subgroups, except for hypertension in the normal BMI group (which could not be calculated because there were only a few subjects with normal BMI and NAFLD, and none of these subjects developed hypertension on follow-up). There was a significant interaction between the smoking status and incident prediabetes/type 2 diabetes and between sex and incident dyslipidemia (**S3 Fig**). In the mediation analysis, NAFLD still showed a significant and direct effect on the incidence of metabolic syndrome, prediabetes/type 2 diabetes, hypertension, and dyslipidemia, independent of the BMI.

## Discussion

We investigated the impact of NAFLD on metabolic dysfunction in a population without obesity and any metabolic syndrome components. We focused on the predictive impact of NAFLD on future incident metabolic syndrome, in isolation from other metabolic confounders. By employing a large general population cohort and non-invasive validated indices to predict the presence of NAFLD, we demonstrated that NAFLD is an early phenotypic predictor of metabolic syndrome, prediabetes/type 2 diabetes, hypertension, and dyslipidemia in a metabolically healthy population. Our results support the concept that NAFLD is not a mere indicator of accompanying metabolic syndrome components but has an independent predictive value for future metabolic syndrome.

### Relationship between NAFLD and metabolic dysfunction

We showed the association of NAFLD with metabolic dysfunction, starting from a metabolically healthy status. In addition to several studies reporting a possible bidirectional relationship between NAFLD and metabolic syndrome [20–24], we provide more strict evidence on NAFLD as an early phenotypic predictor of metabolic dysfunction.

Insulin resistance in adipose tissues, the muscles, and the liver with excessive free fatty acids plays a central role in the development of metabolic syndrome [25]. In this aspect, the liver acts as the site of insulin resistance caused by the overflow of circulating fatty acids. Otherwise, the concept that fat deposition in the liver also contributes to hepatic insulin resistance by the activation of enzymes that impair hepatic insulin signaling [26] and by the altered adipokine secretion in subjects with NAFLD [6] has been suggested. This mechanism could explain our finding of why subjects with intrahepatic fat disposition but without clear evidence of known metabolic syndrome components have a higher incidence of metabolic syndrome. Therefore, NAFLD could be an early surrogate of metabolic disease or a separate disease entity.

### NAFLD occurs upstream during type 2 diabetes development

A high prevalence of prediabetes/type 2 diabetes and increased insulin resistance in patients with NAFLD have been reported [27,28]. Even among euglycemic patients, the incidence of type 2 diabetes was higher in those with NAFLD than in those without [29]. As for the relationship between NAFLD and type 2 diabetes, the direction from NAFLD to type 2 diabetes has recently been highlighted in a meta-analysis [8], and the improvement of NAFLD was associated with the reduction of the rate of incident type 2 diabetes [30].

Similar with previous findings, NAFLD was a significant predictor of prediabetes/type 2 diabetes preceding the development of any metabolic syndrome components in the current study. This suggests that NAFLD is not a mere marker of other shared risk factors in the development of type 2 diabetes but could be involved at the upstream of the developmental cascade of type 2 diabetes. NAFLD may be considered as a surrogate or a possible driver for the progression to prediabetes/type 2 diabetes, although the onset of prediabetes/type 2 diabetes and its relationship with NAFLD may be different among individuals. This result warrants regular screening for prediabetes/type 2 diabetes and early interventions, such as lifestyle modification, in patients with NAFLD even if they do not have any metabolic derangements.

### NAFLD without metabolic dysfunction still predicts future hypertension

Patients with NAFLD had a higher incidence of hypertension than those without NAFLD in several prospective studies [9,31,32]. This is mainly explained by hepatic insulin resistance contributing to endothelial dysfunction and vasoconstriction [33]. In recent studies, the hepatic insulin resistance index correlated with the risk of incident hypertension [34]. Additionally, NAFLD is known to induce dysregulation of the renin-angiotensin-aldosterone system in animal models [2], leading to arterial hypertension.

Despite the clear association of NAFLD with hypertension in prospective studies and its pathophysiological mechanism, it has been difficult to dissect the lone impact of NAFLD on the development of hypertension because NAFLD is accompanied by metabolic dysfunction. Our study is one of the first studies to demonstrate that NAFLD is a significant predictor of future development of hypertension even without overt metabolic dysfunction. Physicians should be proactive in monitoring for the development of hypertension when treating a normotensive subject with NAFLD.

### Reciprocity between NAFLD and lipid metabolism

In general, NAFLD is mainly caused by altered lipid metabolism with increased systemic lipolysis that leads to increased uptake of free fatty acids in and decreased TG export from the liver [35]. After the development of NAFLD, intrahepatic lipid accumulation potentiates further alteration in lipid metabolism, which induces a vicious cycle [35].

In the present study, we proved that “the vicious cycle” could be initiated from NAFLD first, indicating that NAFLD is a predictor of future dyslipidemia. Furthermore, NAFLD has been demonstrated to be associated with decreased levels of circulating HDL_2_ cholesterol, one of the HDL cholesterol subfractions, and increased levels of small, dense low-density lipoprotein cholesterol, both of which are proatherogenic lipid subtypes [36–38]. Therefore, NAFLD should be regarded as one of the risk factors for the aggravation of lipid profiles independent from traditional risk factors, and the association of NAFLD with dyslipidemia might serve as a link between NAFLD and future clinical vascular event by inducing proatherogenic dyslipidemia.

### Significant impact of NAFLD in the patients with low metabolic risks

In the subgroup analysis, female sex was associated with a higher likelihood of metabolic dysfunction. This tendency was also shown in a previous observational study [39]. The result of our study indicates that NAFLD in women should be considered as an equally strong or even a stronger risk factor for metabolic dysfunction, although it is traditionally more prevalent in men. Further investigations as to whether there are sex differences in the prognosis, especially of patients with incident cardiovascular diseases and NAFLD, are warranted, considering that its significance has rarely been discussed.

Furthermore, the condition of the patients with NAFLD with low metabolic risks, such as young age, non-smoking status, and normal BMI, tended to progress more towards metabolic dysfunction. The primary etiology of NAFLD in the low metabolic risk subgroup may include genetic predisposition because the study population had none of the traditional metabolic risk factors. Indeed, there have been efforts to identify both common and rare genetic variants that could explain the development of metabolic syndrome [40]. Therefore, the higher probability of metabolic dysfunction in the low metabolic risk subgroup might indirectly indicate the effect of certain genetic variants on NAFLD, leading to a more rapid disease progression [41].

## Limitations

This study has several limitations. First, although both the HSI and FLI are well-known noninvasive validated indices in the prediction of NAFLD, diagnosis mainly based on these biochemical indices might lead to a certain rate of false-negative or -positive findings. Some limitation of the FLI has also been reported [42], and we did not include ultrasonographic findings in the diagnosis of NAFLD. However, the study populations were limited to subjects without obesity with normal waist circumference, which means that the high scores in both indices were obtained mainly owing to the elevated alanine transaminase and GGT levels. We further excluded heavy drinkers and those with a diagnosis of any liver disease; therefore, it would be difficult to consider a differential diagnosis for the elevation of liver enzyme levels other than NAFLD. Second, the definitions of the outcomes were mainly based on the diagnostic codes, which is an inherent limitation of the study using a claims database. It might cause misclassification or underestimation; however, the methodology based on claims data has been validated in previous studies [43,44]. Third, other metabolic confounders were not included in this analysis. Although detailed family history and additional measurements, such as waist-to-hip ratio or insulin resistance, could make our findings more generalizable, the effect size of the potential confounders would be small because our study population had none of the components of metabolic syndrome and no obesity. Fourth, the extrapolation of the study findings to different ethnic groups may be limited and warrants further investigation in non-Asian populations. Lastly, although the impact of NAFLD according to sex should be considered separately [45], we could not fully analyze the effect according to sex separately owing to the small number of female NAFLD subjectsincluded. This may lead to potential bias, which cannot be adjusted by the multivariate analysis or subgroup analysis performed in the present study. Further, the reproductive status, an important factor for the effect of NAFLD [46], was not accounted for owing to data unavailability in the database.

## Conclusions

In the current study, we demonstrated that NAFLD is an early phenotypic marker predictive of future metabolic disorders. Along with its pathophysiological role regarding insulin resistance in metabolic disorders, NAFLD should be considered as an independent surrogate or even a disease that is associated with future metabolic dysfunction and not a mere hepatic manifestation of metabolic disorders. Our analysis also suggests a possible link between NAFLD and cardiovascular events observed in previous studies [13,14,35]. These results indicate the need for early screening for metabolic disease and earlier planning of therapeutic intervention for patients with NAFLD, which might lead to a reduction of the incidence of cardiovascular diseases caused by metabolic disease with NAFLD in the long term.

## Supporting information

S1 Fig Cumulative incidence of metabolic syndrome in the entire cohort according to the presence of NAFLD. Cumulative incidence of metabolic syndrome in the entire cohort before propensity score matching. NAFLD, Nonalcoholic fatty liver disease.
(DOCX)

S2 Fig Cumulative incidence of each component of metabolic dysfunction in the entire cohort, according to the presence of NAFLD. Cumulative incidence of **(A)** prediabetes/type 2 diabetes, **(B)** hypertension, and **(C)** dyslipidemia in the entire cohort before propensity score matching. NAFLD, Nonalcoholic fatty liver disease.
(DOCX)

S3 Fig Subgroup analysis of each component of the incident metabolic dysfunction. Subgroup analysis of incident **(A)** prediabetes/type 2 diabetes, **(B)** hypertension, and **(C)** dyslipidemia. BMI, body mass index; CI, confidence interval; HR, hazard ratio; NAFLD, Nonalcoholic fatty liver disease.
(DOCX)

S1 Table Proportional change of each metabolic syndrome component between the two health evaluations. The data are presented as number (percentage). HDL, high-density lipoprotein; NAFLD, nonalcoholic fatty liver disease.
(DOCX)

S2 Table NAFLD as a predictor of incident prediabetes/type 2 diabetes, hypertension and dyslipidemia in the entire cohort and the propensity score-matched cohort. Cox proportional-hazards regression analysis to test whether NAFLD would be a predictor of incident prediabetes/type 2 diabetes, hypertension, and dyslipidemia. ^†^, adjusted for age, male, body mass index, current smoking. CI, confidence interval; HR, hazard ratio; NAFLD, non-alcoholic fatty liver disease.
(DOCX)
