# Supplementary material for: Nonalcoholic fatty liver disease is an early predictor of metabolic diseases in a metabolically healthy population
Source: PLoS One. 2019 Nov 4;14(11):e0224626. doi: 10.1371/journal.pone.0224626 (PMC6827890; doi:10.1371/journal.pone.0224626)
Supplement: S1 Fig — Cumulative incidence of metabolic syndrome in the entire cohort before propensity score matching. NAFLD, Nonalcoholic fatty liver disease. (DOCX) [file pone.0224626.s001.docx]

**SUPPLEMENTARY FIGURE**

**S1 Fig.** **Cumulative incidence of metabolic syndrome in the entire cohort according to the presence of NAFLD.**

Cumulative incidence of metabolic syndrome in the entire cohort before propensity score matching. NAFLD, Nonalcoholic fatty liver disease.

**
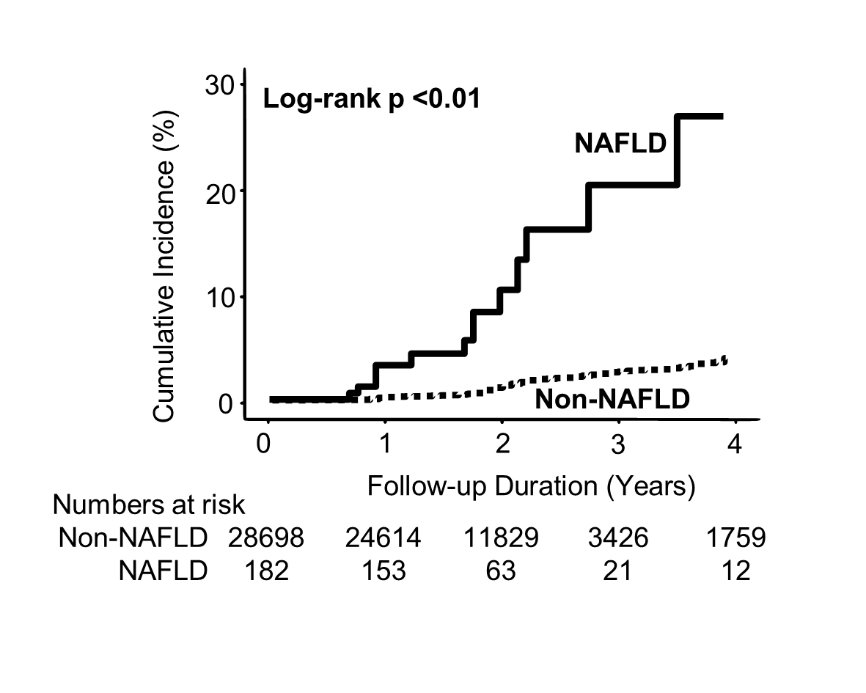
**
