# Supplementary material for: Nonalcoholic fatty liver disease is an early predictor of metabolic diseases in a metabolically healthy population
Source: PLoS One. 2019 Nov 4;14(11):e0224626. doi: 10.1371/journal.pone.0224626 (PMC6827890; doi:10.1371/journal.pone.0224626)

**S2 Fig.** **Cumulative incidence of each component of metabolic dysfunction in the entire cohort, according to the presence of NAFLD.**

Cumulative incidence of **(A)** prediabetes/type 2 diabetes, **(B)** hypertension, and **(C)** dyslipidemia in the entire cohort before propensity score matching. NAFLD, Nonalcoholic fatty liver disease.

**(A)**


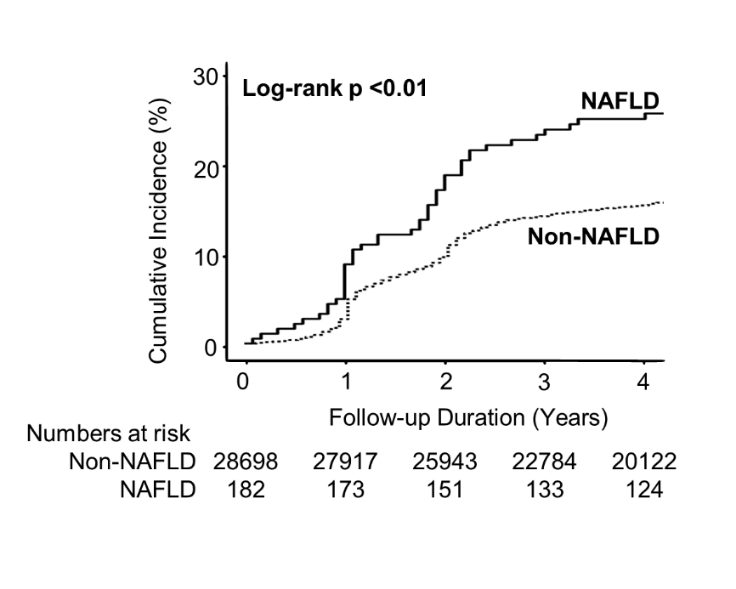


**(B)**


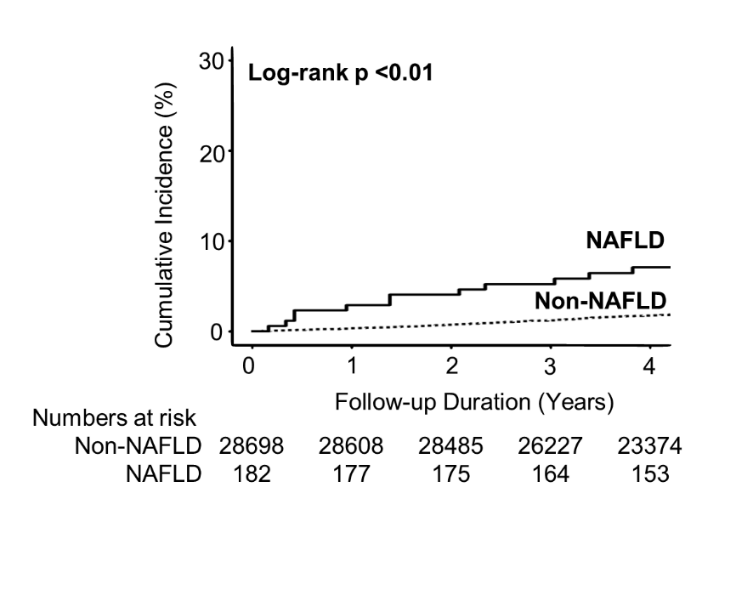


**(C)**


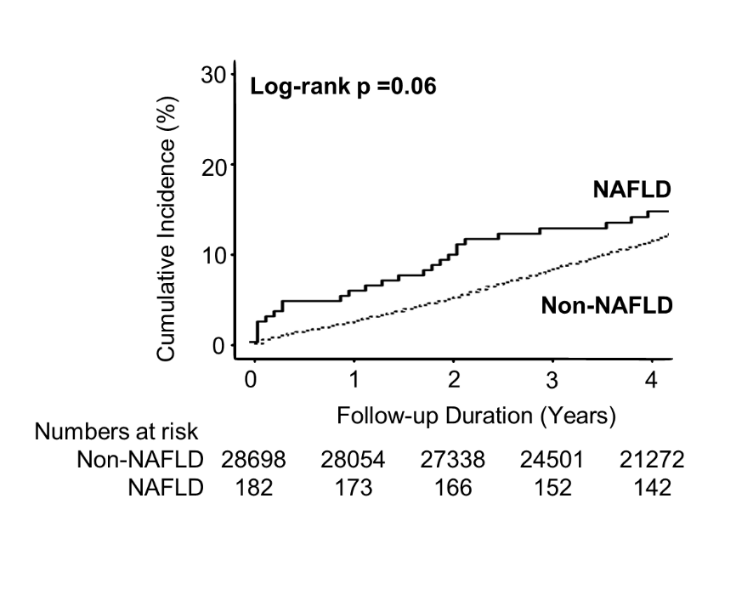

Supplement: S2 Fig — Cumulative incidence of (A) prediabetes/type 2 diabetes, (B) hypertension, and (C) dyslipidemia in the entire cohort before propensity score matching. NAFLD, Nonalcoholic fatty liver disease. (DOCX) [file pone.0224626.s002.docx]
