# Supplementary material for: Nonalcoholic fatty liver disease is an early predictor of metabolic diseases in a metabolically healthy population
Source: PLoS One. 2019 Nov 4;14(11):e0224626. doi: 10.1371/journal.pone.0224626 (PMC6827890; doi:10.1371/journal.pone.0224626)
Supplement: S3 Fig — Subgroup analysis of incident (A) prediabetes/type 2 diabetes, (B) hypertension, and (C) dyslipidemia. BMI, body mass index; CI, confidence interval; HR, hazard ratio; NAFLD, Nonalcoholic fatty liver disease. (DOCX) [file pone.0224626.s003.docx]

**S3 Fig. Subgroup analysis of each component of the incident metabolic dysfunction.**

Subgroup analysis of incident **(A)** prediabetes/type 2 diabetes, **(B)** hypertension, and **(C)** dyslipidemia. BMI, body mass index; CI, confidence interval; HR, hazard ratio; NAFLD, Nonalcoholic fatty liver disease.

**(A)**


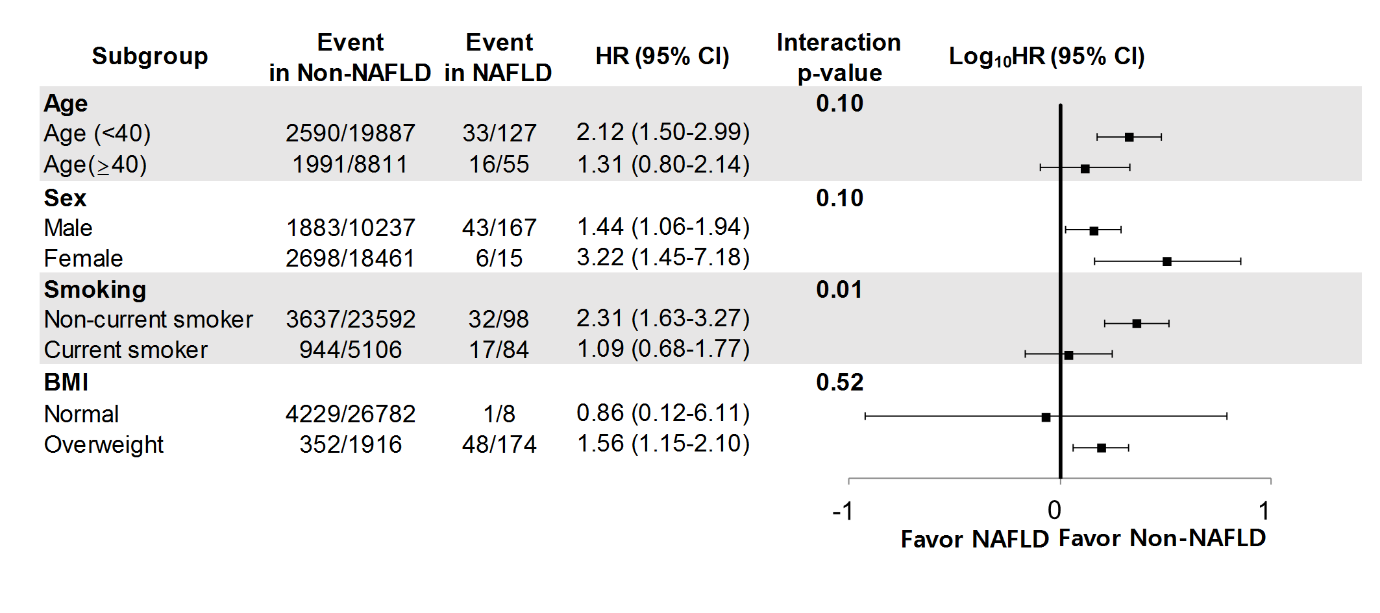
**(B)**

**
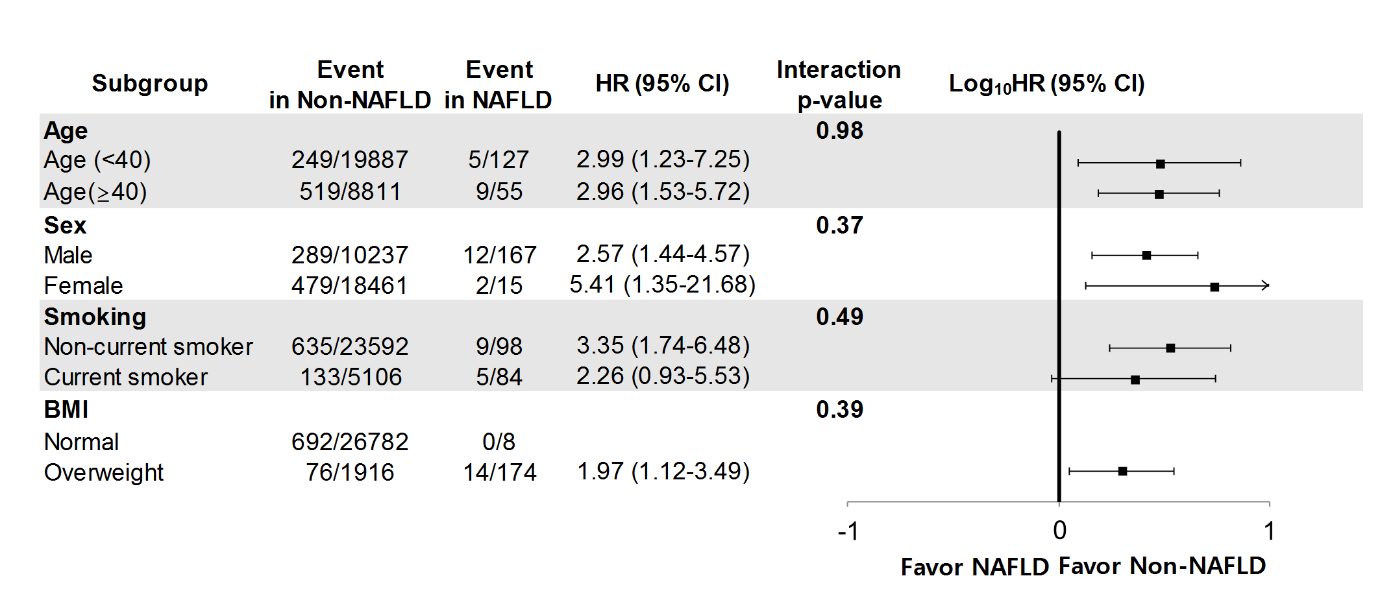
**

**(C)**

**
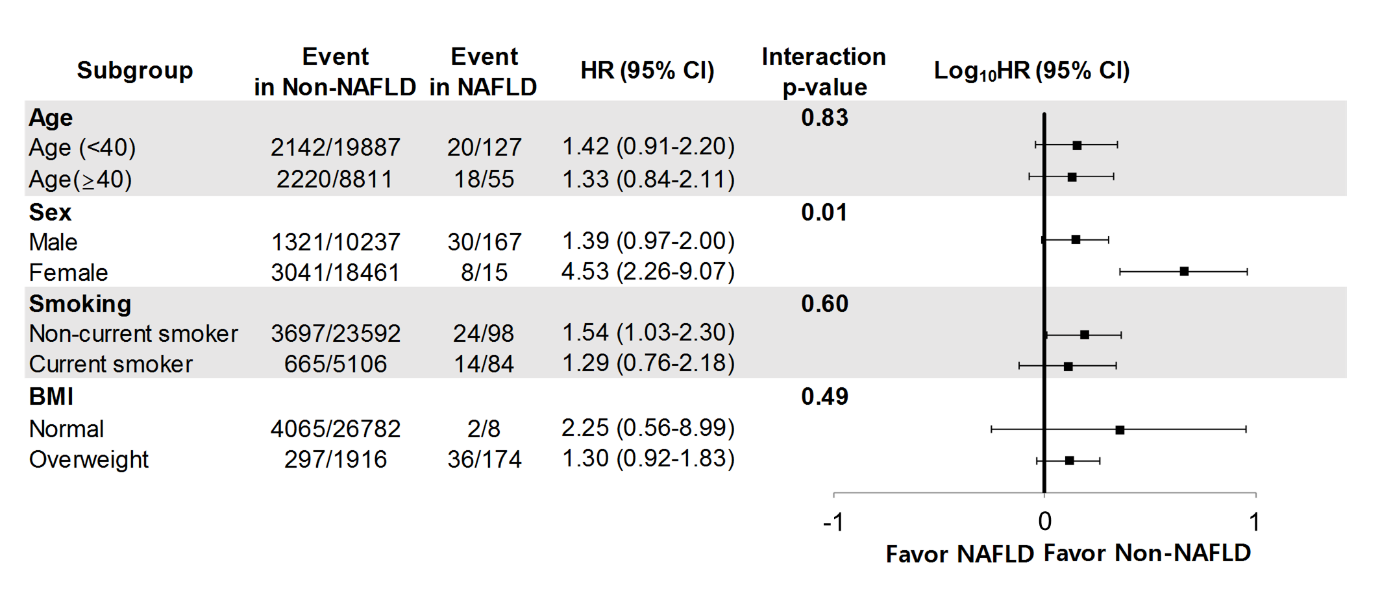
**
