# Supplementary material for: Nonalcoholic fatty liver disease is an early predictor of metabolic diseases in a metabolically healthy population
Source: PLoS One. 2019 Nov 4;14(11):e0224626. doi: 10.1371/journal.pone.0224626 (PMC6827890; doi:10.1371/journal.pone.0224626)
Supplement: S1 Table — The data are presented as number (percentage). HDL, high-density lipoprotein; NAFLD, nonalcoholic fatty liver disease. (DOCX) [file pone.0224626.s004.docx]

**S1 Table. Proportional change of each metabolic syndrome component between the two health evaluations.**

|  | Total cohort (n=28,880) | | |  | | Matched cohort (n=1,092) | | |
| --- | --- | --- | --- | --- | --- | --- | --- | --- |
|  | Non-NAFLD  (n=28,698) | NAFLD (n=182) | p-value | |  | Non-NAFLD  (n=910) | NAFLD (n=182) | p-value |
| **Number of metabolic syndrome components (n, %)** | | | <0.01 | |  | <0.01 | | |
| 0 | 19497 (67.9) | 64 (35.2) |  | |  | 509 (55.9) | 64 (35.2) |  |
| 1 | 7153 (24.9) | 64 (35.2) |  | |  | 276 (30.3) | 64 (35.2) |  |
| 2 | 1754 (6.1) | 39 (21.4) |  | |  | 107 (11.8) | 39 (21.4) |  |
| 3 | 260 (0.9) | 13 (7.1) |  | |  | 16 (1.8) | 13 (7.1) |  |
| 4 | 31 (0.1) | 1 (0.5) |  | |  | 2 (0.2) | 1 (0.5) |  |
| 5 | 3 (0.0) | 1 (0.5) |  | |  | 0 (0.0) | 1 (0.5) |  |
| **Each component of metabolic syndrome (n, %)** | | | | | | | | |
| Waist circumference | 1019 (3.6) | 26 (14.3) | <0.01 | |  | 48 (5.3) | 26 (14.3) | <0.01 |
| Triglyceride | 1907 (6.6) | 65 (35.7) | <0.01 | |  | 130 (14.3) | 65 (35.7) | <0.01 |
| Blood pressure | 3395 (11.8) | 43 (23.6) | <0.01 | |  | 176 (19.3) | 43 (23.6) | 0.22 |
| HDL-cholesterol | 2045 (7.1) | 17 (9.3) | 0.31 | |  | 58 (6.4) | 17 (9.3) | 0.20 |
| Fasting blood glucose | 3214 (11.2) | 39 (21.4) | <0.01 | |  | 134 (14.7) | 39 (21.4) | 0.03 |

The data are presented as number (percentage). HDL, high-density lipoprotein; NAFLD, nonalcoholic fatty liver disease.
