# Supplementary material for: Nonalcoholic fatty liver disease is an early predictor of metabolic diseases in a metabolically healthy population
Source: PLoS One. 2019 Nov 4;14(11):e0224626. doi: 10.1371/journal.pone.0224626 (PMC6827890; doi:10.1371/journal.pone.0224626)
Supplement: S2 Table — Cox proportional-hazards regression analysis to test whether NAFLD would be a predictor of incident prediabetes/type 2 diabetes, hypertension, and dyslipidemia. †, adjusted for age, male, body mass index, current smoking. CI, confidence interval; HR, hazard ratio; NAFLD, non-alcoholic fatty liver disease. (DOCX) [file pone.0224626.s005.docx]

**S2 Table. NAFLD as a predictor of incident prediabetes/type 2 diabetes, hypertension and dyslipidemia in the entire cohort and the propensity score-matched cohort.**

|  | Total cohort (n=28,880) | | | |  | Matched cohort (n=1,092) | |
| --- | --- | --- | --- | --- | --- | --- | --- |
|  | Unadjusted | | Covariate-adjusted^†^ | |  | Unadjusted | |
|  | HR (95% CI) | p-value | HR (95% CI) | p-value |  | HR (95% CI) | p-value |
| Prediabetes/type 2 diabetes | 1.77 (1.34–2.35) | <0.01 | 1.42 (1.06–1.90) | 0.02 |  | 1.97 (1.04–3.73) | 0.04 |
| Hypertension | 2.83 (1.67–4.80) | <0.01 | 2.36 (1.35–4.12) | <0.01 |  | 2.57 (1.35–4.88) | <0.01 |
| Dyslipidemia | 1.35 (0.98–1.86) | 0.06 | 1.49 (1.07–2.06) | 0.02 |  | 1.61 (1.12–2.32) | 0.01 |

Cox proportional-hazards regression analysis to test whether NAFLD would be a predictor of incident prediabetes/type 2 diabetes, hypertension, and dyslipidemia. ^†^, adjusted for age, male, body mass index, current smoking. CI, confidence interval; HR, hazard ratio; NAFLD, non-alcoholic fatty liver disease.
